# Toward a granular molecular-anatomic map of the blood vasculature – single-cell RNA sequencing makes the leap

**DOI:** 10.48101/ujms.v127.9051

**Published:** 2022-10-21

**Authors:** Christer Betsholtz

**Affiliations:** a Department of Immunology, Genetics and Pathology, Rudbeck Laboratory, Uppsala University, Uppsala, Sweden; b Department of Medicine-Huddinge, Karolinska Institutet, Huddinge, Sweden

**Keywords:** Single-cell RNA sequencing, endothelial cells, mural cells, zonation, organotypicity, cell stress

## Abstract

Single-cell RNA sequencing (scRNAseq) marks the birth of a new era in physiology and medicine. Within foreseeable future, we will know exactly what genes are expressed – and at what levels – in all the different cell types and subtypes that make up our bodies. We will also learn how a particular cell state, whether it occurs during development, tissue repair, or disease, reflects precise changes in gene expression. While profoundly impacting all areas of life science, scRNAseq may lead to a particular leap in vascular biology research. Blood vessels pervade and fulfill essential functions in all organs, but the functions differ. Innumerable organ-specific vascular adaptations and specializations are required. These, in turn, are dictated by differential gene expression by the two principal cellular building blocks of blood vessels: endothelial cells and mural cells. An *organotypic* vasculature is essential for functions as diverse as thinking, gas exchange, urine excretion, and xenobiotic detoxification in the brain, lung, kidney, and liver, respectively. In addition to the organotypicity, vascular cells also differ along the vascular arterio-venous axis, referred to as *zonation*, differences that are essential for the regulation of blood pressure and flow. Moreover, gene expression-based molecular changes dictate states of *cellular activity*, necessary for angiogenesis, vascular permeability, and immune cell trafficking, i.e. functions necessary for development, inflammation, and repair. These different levels of cellular heterogeneity create a nearly infinite phenotypic diversity among vascular cells. In this review, I summarize and exemplify what scRNAseq has brought to the picture in just a few years and point out where it will take us.

**Figure:**
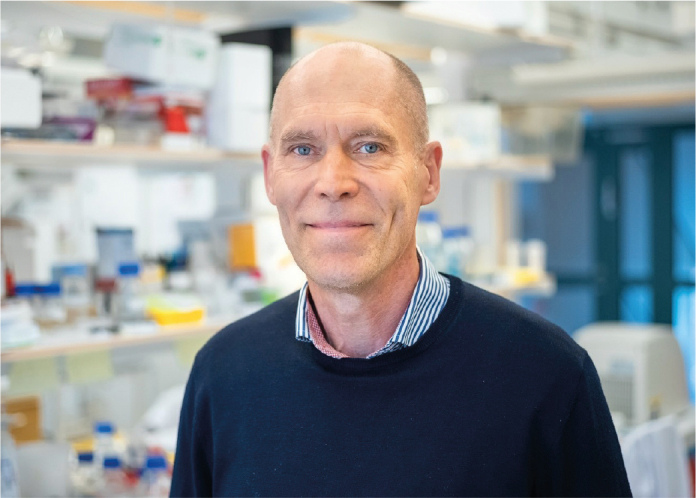
Professor Christer Betsholtz, winner of the Medical Faculty of Uppsala University Rudbeck Award 2020. Photographer: Göran Ekeberg.

## A brief history of vascular anatomy

Blood vessel anatomy has a long history (reviewed in (1)). It has been known since the 2nd century A.D. that arteries and veins emanate from the heart and extend, largely in parallel, through our bodies (Galen of Pergamum (104–210)). For centuries, arteries and veins were believed to carry different fluids: Blood, proposed to nourish the body, was contained within veins, whereas arteries carried blood mixed with ‘spiritous’. One can imagine that the different colors of oxygenated arterial and oxygen-depleted venous blood contributed to this idea.

**Figure 1:**
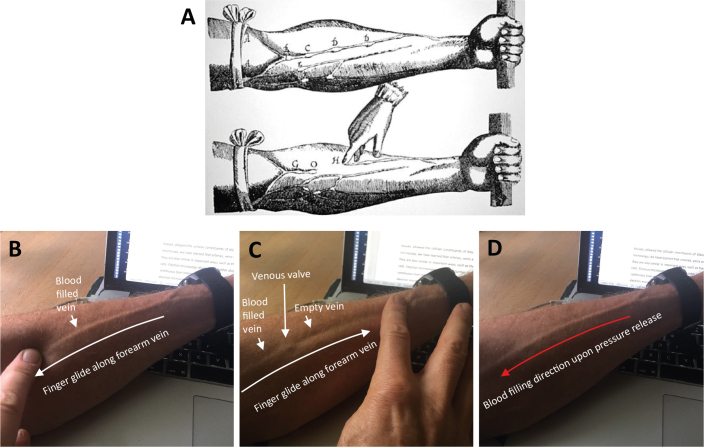
Determination of the blood flow direction in a forearm vein. (A) Illustration from William Harvey’s *Exercitatio Anatomica de Motu Cordis et Sanguinis in Animalibus* (‘De Moto Cordis’, 1628). (B–D) The author repeating Harvey’s experiment while writing this article. (B) The finger was pressed onto the vein at the level of its bifurcation and moved toward the elbow with remaining pressure. The vein remained filled throughout the movement. (C) The finger was pressed onto the vein at the elbow and moved toward the wrist. Blood filling stopped at the venous valve, distal to which the vein was emptied. (D) The finger was removed after the situation shown in C. Blood refilling of the vein visibly occurred from the wrist.

The view that arteries and veins constitute separate containers dominated until the early 17th century with only minor twists. For example, the persian phycisian Ibn Sina (a.k.a. Avicenna 980-1037 A.D.) described arterio-venous anastomoses on the brain surface. Other scholars proposed that some exchange between arterial and venous content might take place through pores in the cardiac septa. This view was questioned by Vesalius (*‘On the Fabric of the Human Body’, 1543*), who among others looked for alternatives. But it was William Harvey (*‘On the Motion of the Heart and Blood in Animals’, 1628*) who, through a series of clever observations, simple experiments, and deductive logic, concluded that blood circulates through a closed vascular system including both the arteries and the veins. Among several key observations, Harvey noted that blood flow direction is opposite in arteries and veins and reasoned that they, therefore, must be connected through invisible conduits in the periphery (reviewed in (2)) (Figure 1).

**Figure 2:**
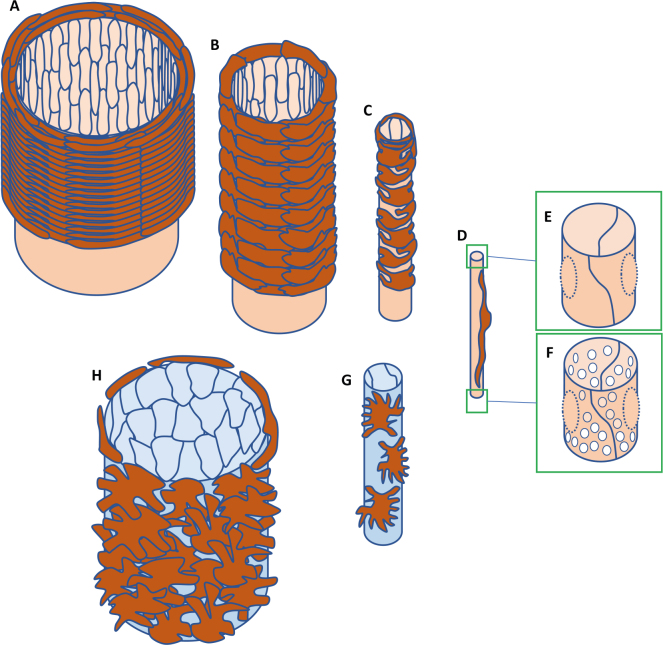
Structural differences between endothelial and mural cell in different types of blood vessels. (A) Elastic arteries display longitudinally oriented endothelial cells and transversally oriented spindle-shaped VSMC, the latter in multiple layers surrounded by elastic matrix. (B) Muscular arteries display longitudinally oriented endothelial cells and transversally oriented spindle-shaped VSMC. (C) Arterioles display longitudinally oriented endothelial cells. VSMCs are not spindle shaped but extend multiple processes transversally encircling the vessel. (D) Arterioles display longitudinally oriented endothelial cells and pericytes. (E) Continuous capillary lacking fenestrations. (F) Fenestrated capillary. (G) Venules display endothelial cells and pericytes without clear orientation in the direction of the blood stream. (H) Veins display endothelial cells and VSMC likewise without clear orientation.

Once microscopy became available, the previously invisible peripheral vascular conduits – capillaries – could be observed. Through the 19th and 20th centuries, histology, the study of the microscopic structure of tissues, allowed the cellular constituents of blood vessels to be investigated. Using light- and electron microscopy, we learned that arteries, veins, and capillaries are in part differently constructed, although also similar, e.g. in the luminal lining by endothelial cells (Figure 2). Electron microscopy further showed that the blood vessel endothelial lining is either continuous (non-interrupted) or discontinuous (displaying gaps) in a vessel-type and organ-specific way. Discontinuous vessels were, for example, found in liver and spleen, part of the kidney vasculature and in endocrine organs (3–5). Here, pores, or *fenestrae*, with diameters of 50–300 nm were found to go *through* the endothelial cells, but gaps between endothelial cells were also noted in inflammatory situations (6, 7).

All blood vessels were found to contain two principal cell types: endothelial cells and mural cells. The mural cells coat the endothelium to a variable degree in area and thickness. Mural cells encompass vascular smooth muscle cells (VSMCs) and pericytes. VSMCs reside in elastic arteries, muscular arteries, arterioles, and veins and vary in their phenotype depending on the vessel type. Pericytes are instead found in capillaries and venules. The largest vessels harbor yet an additional cell type, fibroblasts, which, together with nerves and supporting capillaries, *vasa vasorum*, reside in the tunica adventitia, the outermost vascular tissue layer. The morphological diversity of mural cells ranges from the spindle-shaped VSMC present in arteries to the irregular-shaped VSMC of veins as well as the thin slender pericytes of capillaries and venules (8) (Figure 2).

Whether molecular and functional differences exist between endothelial and mural cells of different vessels could, however, not be concluded from histological investigations. Molecular heterogeneity was instead suggested by the differential distribution of singular makers, often discovered by chance. Early examples include Bmx and Ephrin-B2, which were identified as arterial (9, 10) and Eph-B4 and Coup-TFII as venous endothelial markers (10, 11). But any deeper insight into the molecular heterogeneity of vascular cells was largely lacking before single-cell RNA sequencing (scRNAseq). Microscopy of live specimen revealed functional differences, such as the trans-endothelial migration of leukocytes, which takes place mainly in venules, suggesting localized expression of leukocyte adhesion molecules.

## A brief history of scRNAseq

Single-cell sequencing was elected ‘Method of the year’ in 2013 by *Nature Methods* (12). While in theory, this technology held tremendous promise already before 2013, it took a several years for the methodology to mature sufficiently to allow deep (thousands of genes) single-cell transcriptomes to be obtained from thousands of cells in the same experiment, thereby offering detailed transcriptomic maps of complex organ such as the brain (13). Based on scRNAseq, the traditional classification of cell types (based on cell morphology and a few cell-type-selective markers) could now be revisited into a taxonomy based on genome-wide quantitative gene expression data. This work is in progress. Emerging cellular taxonomies appear to reflect scientists’ urge to present something new and are, therefore, still somewhat ‘wild west’. Undoubtedly, the area will mature as more data collect and get reproduced. Currently, single-cell transcriptomic atlases have been generated for several organs in mouse (14), rat (15), and human (16), as well as for some organs also in non-mammals such as zebrafish (17), using different scRNAseq techniques. To-date, more than 100 different techniques for single-cell ‘omics’ have been published (*‘Single-Cell-Omics.v2.3.13 @albertvilella’*).

**Figure 3:**
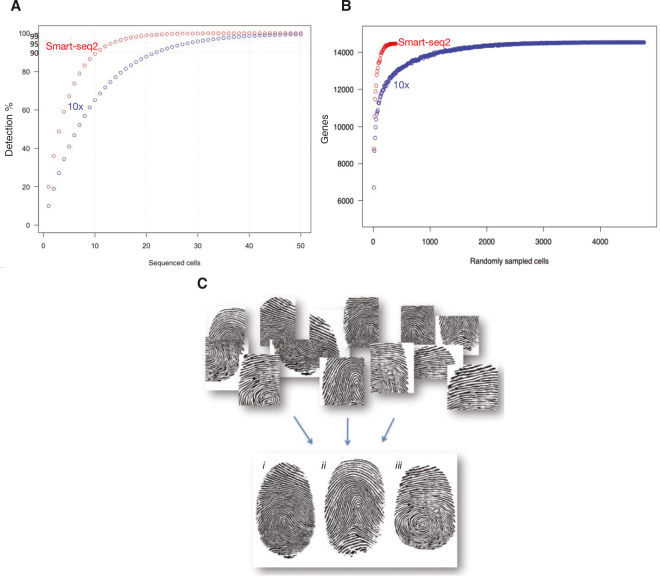
Single-cell RNA sequencing (scRNAseq) in numbers and an analogy. (A) An oversimplified theoretical model of scRNASeq gene detection using droplet sequencing (10x), capturing 10%, and Smart-seq2, capturing 20% of the mRNA molecules in a cell. Assuming that all genes are equally abundant in the cell (which is not true; the abundance is highly variable), and each single-cell randomly contributes *P* (percentage) of the genes expressed by that cell type, and then by sequencing 1 cell, *1–P genes* will not be found and *P genes* will be identified, and by sequencing 2 cells, (*1–P)^2 genes* will not be found and *1–*(*1–P)^2 genes* will be identified, *etc.* By sequencing n cells, (*1–P)^n genes* will not be found and *1–*(*1–P)^n genes* will be identified. As shown in the graph, ≈ 99% of the genes will be detected though sequencing ≈ 20 cells using Smart-seq2, whereas it takes ≈ 50 cells to reach the same depth using 10x. (B) Data from a real experiment, in which the same cell type (brain capillary endothelial cells from adult mouse brain) was analyzed by scRNAseq using Smart-seq2 or 10x. In the Smart-seq2 analysis, ≈ 400 cells were sequenced, and in the 10x analysis, ≈ 4,800 cells were sequenced. The graph illustrates that Smart-seq2 detects more genes per cell (10,000 genes require ≈ 10 cells to be sequenced by SmartSeq2 and ≈ 40 cells to be sequenced in a 10x experiment) as well as overall in the compiled data (≈ 14,500 genes in both the Smart-seq2 and 10x experiments). (C) The fingerprint analogy. As in forensic fingerprint analysis, two non-overlapping print fragments may be connected through serially nested overlaps with other fragments and concluded to derive from the same finger.

In spite of the huge technological diversity, the currently used protocols for scRNAseq are basically two (18, 19): The dominating one is based on encapsulation of single cells in liquid droplets together with beads that contain primers for complementary (c)DNA synthesis (20). Thanks to bead-specific DNA sequences (‘bar codes’) that get incorporated into the cDNA, many (optimally ≈ 5,000) single cells may be sequenced simultaneously and the individual sequences back-tracked to single droplets/cells using the bar-code. The second technique relies on single-cell sorting, usually by fluorescence-activated cell sorting (FACS), into microliter volumes, in which cell lysis and cDNA synthesis take place. This methodology also takes advantage of incorporated cDNA ‘bar-codes’ to allow cost-effective simultaneous sequencing of multiple cells, usually ≈ 100–400, depending on the type of microtiter plate used. While this is 10–50-fold less compared to droplet sequencing, an increased sequencing depth per cell may be achieved when methods such as SmartSeq2 (SS2) are used (21). For comparison, a standard scRNAseq experiment using the droplet technique provides sequence reads from around 10% of the expressed genes in a single cell, compared to around 20% for FACS-SS2 (Figure 3).

Is this important? And which method should one use? The answers depend on the scientific question. The droplet technique is commercially available, requires a minimum of instrumentation, and is less expensive overall. Hence, droplet sequencing is favorable when common cell types are explored and high cell numbers are needed. Droplet sequencing has, therefore, so far been advantageous in the scRNASeq analysis of whole organs or organisms. An updated cell atlas of the mouse brain, accomplished by droplet sequencing of around 500,000 cells (22), is one of several examples. However, when the target cells are rare (such as stem cells), and if a method exists for their isolation by FACS, the FACS-SS2 protocol may be preferable for at least two reasons: 1) there is no lower limit to the number of cells that can be handled (which is the case with droplet sequencing), and 2) deep information is obtained from each cell. In addition, because cells are often injured during FACS – which may skew the cell’s mRNA profile if the cell survives – it helps if cells get sorted directly into lysis buffer, as opposed to being collected as a concentrated cell suspension for subsequent droplet encapsulation. The latter takes time and may lead to injury-induced transcriptional responses. As further discussed below, some skewing of the original *in vivo* transcriptome is anticipated in all scRNAseq experiments. However, if recognized, the problem may be handled to prevent erroneous interpretation of the data.

As mentioned, the mRNA capture from a single cell is incomplete (10–20%) and stochastic, and, hence, the transcriptomes obtained from any two identical single cells are notably not identical. Therefore, partial overlaps between several single-cell transcriptomes are required to compute the full transcriptome of a cell type. This requires clustering algorithms, which are nowadays part of any standard package of computational methodology for scRNAseq. The clustering principle may be illustrated by its analogy to forensic analysis of fingerprints, in which many fragments of prints collected from multiple surfaces get assembled into full fingerprints by computational image analysis (Figure 3). Likewise, the compiled transcriptomes of cells that cluster together can be used to construct the complete transcriptome for a particular cell type, subtype, or state. Only a small amount of mRNA sequence information is usually required for the determination of the general cell class (e.g. hepatocyte, cardiomyocyte, and neuron). However, genome-wide quantitative transcriptomic information obtained by compiling partial transcriptomes from multiple identical cells allows closely related cell subtypes (e.g. different T-lymphocytes), states of cell activation (e.g. differentially activated macrophages), or proliferation (e.g. stage in the cell cycle, see further later) to be distinguished.

In summary, scRNAseq is a game changer in life science because it allows cellular heterogeneity to be decoded into genome-wide quantitative gene expression information.

## Previous methods for the systematic transcriptional analysis of cells

Bulk RNA-seq, introduced in the mid 2000s, was largely made possible through the development of Next Generation Sequencing. Before bulk RNA-seq, DNA microarrays for multiplex nucleic acid hybridization had been invented by Pat Brown in the 1980s and applied to multiscale gene expression analyses (23). In the early 2000s, our own laboratory began generating spotted cDNA microarrays for vascular gene expression mapping. We printed collections of partial-length cDNA clones (expressed sequence tags, EST), annotated against available genome assemblies, onto multiples of glass slides, which were subsequently hybridized against cDNA prepared from different tissues. In this way, genes with stronger hybridization against mRNA prepared from vascular compared to non-vascular tissues could be listed as candidate vascular-specific genes. We searched, for example, for new markers of brain pericytes (24, 25) and kidney glomerular capillaries (26). These and other studies significantly expanded the repertoire of markers for endothelial cells and pericytes, but because cell ‘bulks’ were analyzed, the obtained information represented an average; any intrinsic variation caused by heterogeneity among the intended cells, or the presence of non-intended (contaminating) cell types, were invisible. The findings were also not always what we expected. For the kidney glomerulus, we anticipated to find genes specific for glomerular endothelial cells but found mostly markers of podocytes (glomerular epithelial cells) (26). In retrospect, this result was anticipated because podocytes are more distinct (from the other cells in the kidney) than the glomerular endothelial and mural (mesangial) cells.

## ScRNAseq identifies cell subtypes and states

**Figure 4:**
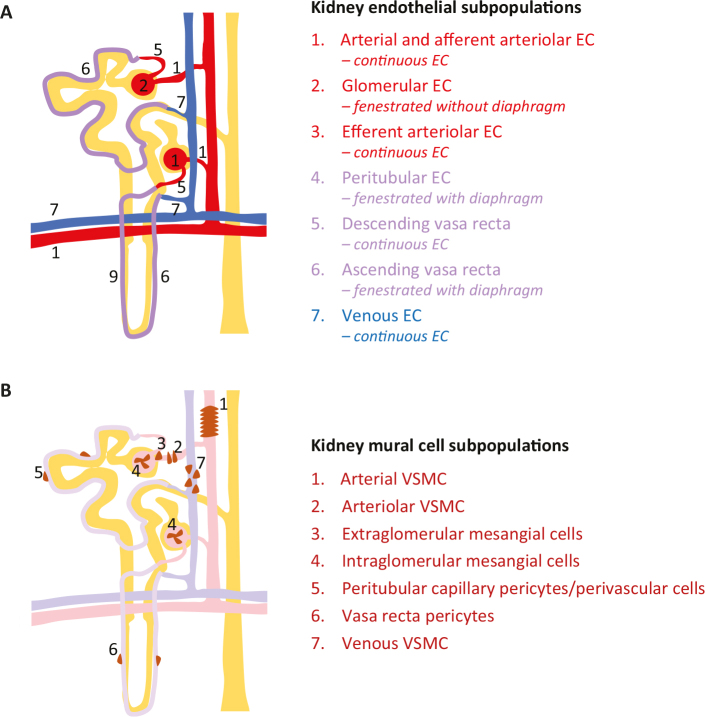
Vascular cell heterogeneity in the kidney. (A) Seven different endothelial phenotypes resolved by single-cell RNA sequencing (scRNAseq) (27, 28) correspond to 7 different locations of the vessels and their ultrastructural characteristics (fenestrated or continuous) along the nephron (yellow). (B) Seven different locations of mural cells along the nephron. So far, scRNAseq has clarified the different gene expression pattern of intraglomerular mesangial cells in comparison with extraglomerular mesangial cells and/or VSMC of the afferent and efferent arterioles (29).

ScRNAseq analysis of whole kidney was recently used to uncover molecular differences between the endothelial cells in close apposition to different parts of the nephron (27, 28) (Figure 4). ScRNAseq analysis of isolated glomeruli also revealed heterogeneity, in this case between the vascular cells located inside (intraglomerular) as opposed to those immediately outside of the glomerular tuft (juxtaglomerular) (29, 30). These heterogeneities would not have been captured by bulk RNAseq and, therefore, illustrate the power of scRNAseq, but they also stress the necessity of a two-step procedure to provide tissue context. Following scRNAseq analysis (step 1), *in situ* gene/protein analysis of differentially expressed genes (step 2) is required to put the cell type/subtype into an anatomic map. Methodology referred to as spatial transcriptomics (31) has been developed for the sampling of transcriptional information directly from tissue sections, thereby providing a direct anatomic context to the transcriptomic information. However, these methods are too low in spatial resolution to distinguish between closely apposed cells such as endothelial cells and pericytes. Both these cells are extremely thin, and they are firmly attached to each other by a joint basement membrane, making them hard to resolve even with high-resolution light microscopy. Rather than replacing scRNAseq, spatial transcriptomics should be viewed as a complement offering higher level information, such as regional cell type distribution within a large organ such as the brain (32, 33).

### Vascular zonation

**Figure 5:**
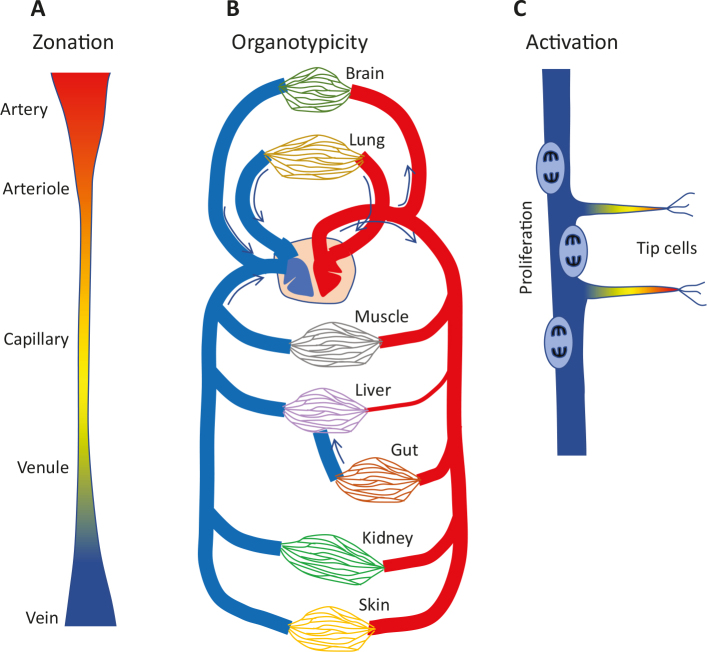
Three levels of endothelial heterogeneity *in vivo*. (A) Zonation along the arteriovenous axis. Single-cell RNA sequencing (ScRNAseq) suggests seamless transitions between gene expression phenotypes. (B) Organotypicity is mainly a microvascular specialization designed to match the specific organ function. For capillary endothelial cells, known organotypic features include type and tightness of intercellular barriers, presence or absence of fenestrations, expression levels of transporters, e.g. for lipids, amino acids, sugars, metal ions, and hormones, and receptors for growth factors and hemodynamic regulators. Mural organotypicity (not illustrated) has been demonstrated in pericytes (34) and muscular arterial VSMC (47) but is currently less explored than endothelial organotypicity. (C) Activation. The illustration shows angiogenic sprouting and cell proliferation. Other types of endothelial activation include changes in permeability and responses to endogenous and exogenous inflammatory mediators.

One of the insights from our scRNAseq analysis of the adult mouse brain vasculature was that vascular cells display zonation (34, 35). In biology, this term is used to implicate a gradual change in cellular phenotype along an anatomic axis. Our data suggested that endothelial cells, indeed, change their gene expression gradually along the arterio-venous (A–V) axis (34) (Figure 5A). We observed zones of gene expression at differential levels for a large number of genes with different nested patterns, which when combined together suggested seamless transitions from one cell phenotype into the next. For brain mural cells, the zonation pattern was different. For example, an abrupt transition in phenotype from one cell to the next was observed in the terminal arterioles, where VSMC expressing mRNA coding for a smooth muscle contractile machinery was followed by pericytes lacking the SMC contractile machinery and instead expressing pericyte markers. Notwithstanding the biological importance of these different modes of zonation (an endothelial continuum *vs.* a punctuated zonation of mural cells), these data raise two important perspectives. First, mapping of the molecular underpinnings of vascular zonation requires scRNAseq; no other currently available technology has the ability to detect it. Secondly, a cell type/subtype concept based solely on transcriptomics will inevitably be ambiguous. Most vascular biologists would probably agree to a taxonomy in which blood vascular endothelial cells are seen as a *cell type* (within the larger *class* of endothelial cells also encompassing lymphatic endothelial cells and endocardial cells). Arterial, capillary, and venous endothelial cells, on the other hand, would be viewed as *subtypes* of endothelial cells. However, the molecular zonation along the A–V axis shows that arterial, capillary, and venous endothelial subtypes are not homogeneous entities but rather a continuum of phenotypes (Figure 5A). If a distinguishable transcriptome reflects a cell subtype/state, the observed A–V zonation would imply an infinite number of endothelial cell subtypes, which is not meaningful. At present, endothelial zonation has been analyzed in only a few organs, including the brain (34) and the liver (36, 37). Additional examples, including cross-organ and cross-species comparative analyses, will be needed before a useful and widely applicable taxonomy for vascular cell A–V subtype designation may be adopted.

### Organotypicity

If A–V zonation provides one level of vascular cell heterogeneity, organotypicity is another. Organotypicity refers to the organ-specific adaptation and specialization of the vasculature, matching each organ’s physiological functions and need (38, 39) (Figure 5B). The kidney example above demonstrates intra-organ vascular differences, but the differences between organs are even greater. For example, the blood-brain barrier (BBB) is, by and large, an organotypic specialization of the brain’s endothelial cells, providing them with a tightly regulated selectivity for trans-endothelial diffusion and passive and active transport of solutes and large molecules (40).

**Figure 6:**
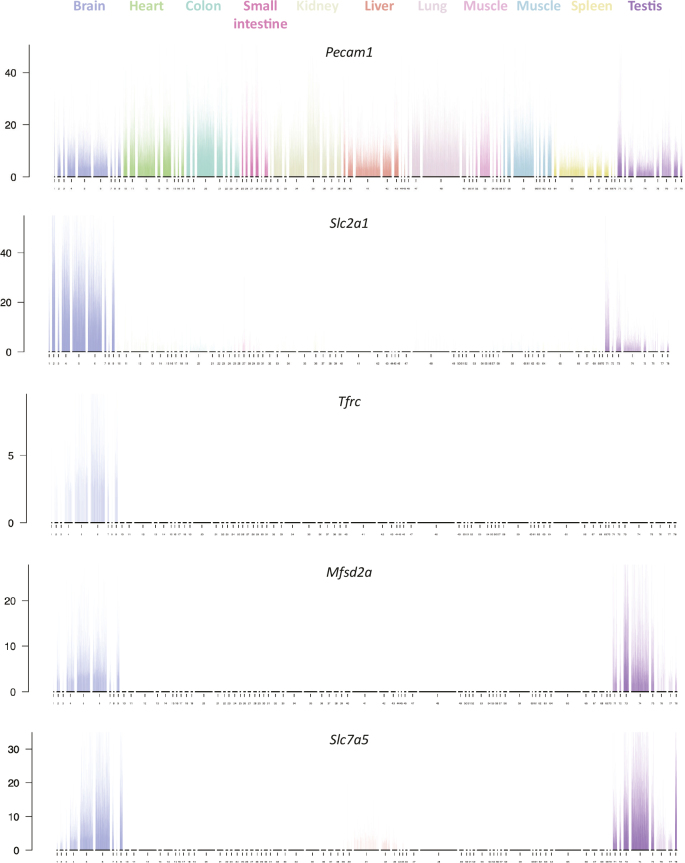
Organotypic endothelial gene expression. The figure shows an *in silico* experiment that assesses the expression of five genes in endothelial single-cell RNA sequencing (scRNAseq) data from 11 organs (data extracted from (41)). For each organ, multiple endothelial clusters (subtypes) were identified, and in total, 78 clusters were reported. *Pecam1* is a ubiquitous endothelial cell marker, whereas *Slc2a1, Tfrc, Mfsd2a*, and *Slc7a5* have been considered brain-specific endothelial transporters. However, the data show that only *Tfrc* is exclusively expressed in brain, whereas the others are also expressed both brain and testis. Data are displayed as bar plots where each cell is represented by a fixed position on the x-axis and the expression level as normalized RNA sequence counts on the y-axis. Note that *Slc7a5* is also expressed (weakly) in liver endothelial cells. X-axis: cluster numbers. Y-axis: normalized sequence counts.

To reliably study vascular organotypicity by scRNAseq, data from different organs need to be generated using the same technical platform, and, to avoid batch effects, preferably also by the same investigators. In our 2018 study, we demonstrated organotypicity for endothelial cells and pericytes by comparing them between brain and lung (34, 35). Subsequent work from Kalucka et al. analyzed adult endothelial cells from 11 organs in the adult mice, confirming organotypic gene expression patterns across all (41). Importantly, published data like these allow *in silico* investigations on individual gene expression patterns, in the case of Kalucka et al. for more than 12,000 genes across 78 endothelial subtypes/states in 11 organs, altogether about 1 million datapoints (1 datapoint = the average expression of a gene in one endothelial subtype). Figure 6 shows the outcome of one such experiment. Here, the expression of four plasma membrane molecular transporters, all widely assumed to be BBB-specific, is compared: the glucose transporter Glut1 (*Slc2a1*), the transferrin receptor (*Tfrc*), the omega 3 fatty acid transporter (*Mfsd2a*), and the essential amino acid transporter Lat1 (*Slc7a5*). The experiment shows that out of the four BBB-specific genes, only *Tfrc* is truly brain endothelial-specific. *Slc2a1*, *Mfsd2a*, and *Slc7a5* are all highly expressed also in testicular endothelial cells. The testis is known to display a barrier to circulating large molecules – the blood-testis barrier – through the expression of tight junctions between Sertoli cells (42). However, to what extent the testis endothelial cells contribute to this barrier is unknown. Similar to neurons, gonadal cells likely need protection from toxic and mutagenic xenobiotics occasionally taken up into the blood. The blood-testis barrier likely explains the need for specific machinery that facilitates uptake of nutrients, and part of this machinery appears to reside in the endothelium.

As an organotypic vascular specialization, the BBB deserves further commenting: It is not a singular phylogenetic invention but has evolved independently several times and exists also beyond the vasculature. For example, insects have a barrier referred to as a BBB that encapsulates the brain, in spite of lacking both blood and regular blood vessels. The insect BBB is localized to glial cells connected with tight junctions separating the central nervous system (CNS) from the surrounding milieu, i.e. the insects’ open circulatory system containing hemolymph (reviewed in (43)). Interestingly, also mammals have glial cells (i.e. the astrocyte end-feet) that border the brain’s surface and blood vessels, but these lack tight junctions, which are instead present in the endothelial cells. In sharks and skates, however, perivascular astrocytes display tight junctions, whereas the brain vascular endothelial cells do not (reviewed in (43)). These examples show that fundamental aspects of vascular organotypicity may differ between species, a relevant consideration when animals are used as models of human diseases. For example, it remains to be determined to what extent mice are good models for human BBB functions, pathological disturbance, and brain neuropharmacology. The detailed scRNAseq comparison between the mouse and human BBB will be informative in this regard but remains to be done. Vascular scRNAseq will also bring increased understanding of species-specific differences in the physiology of other organs. A recent example comes from the mammalian lung, in which alveolar capillaries have been shown to contain two different endothelial cell types: one – aerocytes – that forms the respiratory surface together with type-I alveolar epithelial cells, and another cell possibly regulating vasomotor tone and capillary regeneration (44, 45). This type of lung alveolar endothelial cell specialization was documented in several mammals but was missing in reptiles (45).

These examples demonstrate the unique power of scRNAseq to provide information about endothelial organotypicity. Decades of reductionistic work will now be required to disentangle the nature and details of implicated functional variations and their evolution. The organotypicity of mural cells is currently much less investigated than the organotypicity of endothelial cells (46). The differences found between brain and lung pericytes (34) suggest that molecular transport is a major organotypic trait in pericytes similar to endothelial cells. Moreover, our recent study of VSMC demonstrates organotypicity among arterial VSMC related to, for example, the expression of extracellular matrix components and regulators of ion fluxes (47).

### Cell activation

**Figure 7:**
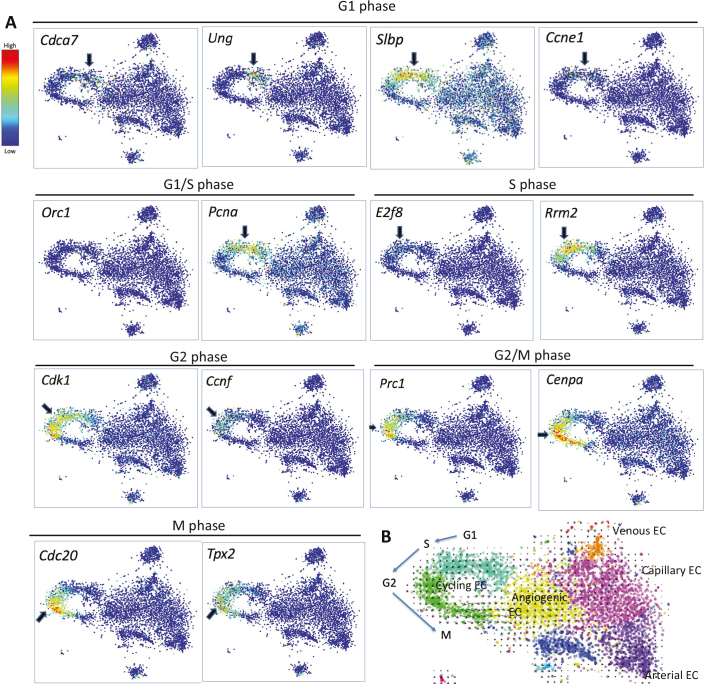
Cell states assessed by single-cell RNA sequencing (scRNAseq). (A) Endothelial cells from developing mouse heart displayed by *t*-stochastic neighbor embedding (*t*-SNE). Cells, marked as dots in the graphs, that have similar transcriptomes (as determined by scRNAseq) are located close to each other. Expression of the indicated cell cycle markers (suggested from literature) is shown as color heat (red = high; blue = low). (B) The same *t*-SNE map with a superimposed RNA velocity analysis (arrows) (55) confirms the direction of progression in the cell cycle suggested by the markers. The cell clusters are colored and partially annotated. The data suggest that capillary endothelial cells become activated, and from their activated stage, they enter and exit the cell cycle. Data were extracted from (56).

A third type of gene expression heterogeneity reflects the state of cell activation (Figure 5C). For endothelial cells, this includes physiological and pathological angiogenesis, inflammatory activation, and disease-associated loss of organotypicity, all characterized by cellular heterogeneities, which, prior to scRNAseq, were approachable only by analysis of singular markers. Similar to A–V zonation, the heterogeneous nature of cell activation gets ‘lost’ in the average of bulk analyses. ScRNAseq analyses of the vasculature in different diseases are now being published at an increasing pace (48–51). Our own work provides some examples along these lines. Using scRNAseq, we found heterogeneous changes in extracellular matrix production and loss of BBB-markers in endothelial cells in human glioblastoma (50). Of note, however, the BBB-marker loss was only partial, even in the most extensively pathologically altered endothelial cells. Residual organotypic expression included drug efflux transporters, suggesting that glioblastoma cells may be protected against chemotherapeutic agents by the tumor endothelial cells in spite of gross evidence for macromolecular leakage in the tumor (50). We have also used scRNAseq to analyze changes in endothelial cell states that take place in mouse brains with pericyte hypoplasia and a resulting BBB dysfunction (52, 53). This model displays extensive vascular heterogeneity at both morphological and functional levels; for example, angiogenic sprouting and microaneurysms formation. ScRNAseq analysis likewise revealed heterogenic changes in the endothelial cells, and by linking the expression of individual marker genes and proteins to distinct vascular morphologies, genome-wide transcriptomes of distinct endothelial states such as tip cells and microaneurysms could be established (53). Tip cells were originally described as non-proliferating endothelial cells emanating numerous filopodia toward sources of vascular endothelial growth factor (54), and the scRNAseq data from pericyte-deficient mice confirmed that tip cells were, indeed, non-proliferative (53). Endothelial cell proliferation, on the other hand, may be studied by scRNAseq with unprecedented precision. Figure 7 shows a t-SNE map of endothelial cells from the early postnatal heart, in which not only proliferation as such but also the individual cells’ position in the cell cycle may be determined.

## Methodological caveats under the magnifying glass

Which genes are expressed and which genes are not expressed, by a cell? The answer to this simple question is critical to both the physiological functions of the cells and the cell taxonomy. Earlier attempts to molecularly map the BBB and other vascular organotypicities by comparing bulk-isolated cells have been performed (57–59), and at first glance, these approaches seemed unobjectionable due to the ease by which individual organs can be separated for molecular analysis. However, the dissociation of an organ into single cells and the subsequent sorting of cells of a particular type such as endothelial cells are methodologies burdened with multiple ambiguities and caveats. In fact, scRNAseq has taught us that even the most refined protocols for tissue dissociation result in cell suspensions of mixed quality and purity.

Therefore, when bulk isolates of endothelial cells are compared between organs, several confounders need to be taken into consideration, including not only intra-organ vascular heterogeneity, as described above for the kidney, but also contamination by non-endothelial cells. For example, bulk isolated brain endothelial cells are notoriously contaminated with fragments of pericytes, which thereby contribute pericyte transcripts to the ‘endothelial’ transcriptome in the proportion of the size of contamination (60). Moreover, by searching the transcriptomes of bulk-isolated endothelial cells from two different organs for organotypic differences, it is common to identify contaminating mRNAs emanating from other organ-specific cells. For example, the comparison between endothelial cells from brain and heart may end up listing also neuronal and cardiomyocyte-expressed genes. Contamination is an often-underestimated confounder in transcriptomics.

### Contamination in scRNASeq data

**Figure 8:**
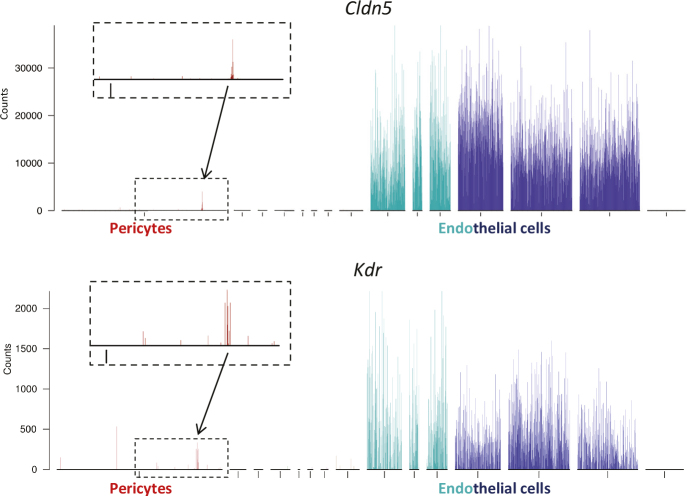
Cell contamination assessed by single-cell RNA sequencing (scRNAseq). The graphs show bar plots of two known endothelial-specific genes: the tight junction gene *Cldn5* and the vascular endothelial growth factor receptor-2 gene *Kdr*. Besides their abundant representation in endothelial cells, they are also found in a small number (≈ 1%) of pericytes (magnified in the insets). The graphs are from (34). Analysis of other endothelial markers shows similar results (34). Pericytes containing endothelial transcripts are most likely contaminated by endothelial cell fragments that contribute endothelial mRNA in proportion to the size of the contaminating fragments.

An alternative to scRNAseq is single-nucleus (sn)RNAseq, which, in theory, should eliminate the problem of cell fragment contamination, since cells fragments are presumed to attach by their cell membranes. However, snRNAseq has other problems. The disruption of cells preceding the nuclear isolation creates a ‘soup’, in which mRNA from one cell may bind to, and even taken up by, a nucleus of another cell. Indeed, cardiomyocyte-specific transcripts were found to be abundant in endothelial nuclei from the heart, suggesting profound ambient RNA contamination (61). Although both ambient and cell fragment-contributed mRNA contamination may confound also scRNAseq data, the nature of scRNAseq makes it possible to identify individual contaminated cells and the contaminating sources (cell types), and handle (e.g. by *in silico* removal of the contaminated cells) and re-analyze the data accordingly (Figure 8). This exercise is not possible with other methods, including bulk RNAseq where the contamination becomes an invisible part of the average.

A recent example shows that a careful analysis of contamination in scRNAseq data provided important information related to COVID-19-associated endotheliitis. It has been suggested that endothelial cells get directly infected by SARS-CoV-2 through binding of the virus to the receptor Angiotensin converting enzyme 2 (ACE) present on endothelial cells (62, 63). ACE2 expression by endothelial cells had, indeed, been suggested by immunostaining (64) seemingly supported by scRNAseq data (65, 66). However, the endothelial ACE2 expression was low, and ACE2 reads found only in a proportion of the endothelial cells. ScRNAseq further suggested that ACE2 expression was high in cardiac pericytes (66). By comparing the minority population of heart endothelial cells positive for ACE2 with the majority population of endothelial lacking ACE2, we found that the former invariably carried multiple markers of pericytes (67). The 50 top-correlating transcripts with ACE2 in endothelial cells were all abundant in pericytes, and many of them were already known as pericyte markers. We, therefore, concluded that the ACE2, observed in a small proportion of the endothelial cells, must have been contributed by contaminating fragments of pericytes attached to the endothelial cells. These conclusions were confirmed in a subsequent study in the mouse, in which we found that Ace2 (mouse ACE2) is not expressed by endothelial cells in any organ, but instead organotypically by pericytes in heart and brain, leading to contamination of a small proportion of the endothelial cells in these organs (68). Reciprocal contamination by neighboring cells must, therefore, always be considered as a possible confounder in scRNASeq data. Figure 8 illustrates the opposite situation: pericytes contaminated by endothelial cell fragments.

### Cell stress

**Figure 9:**
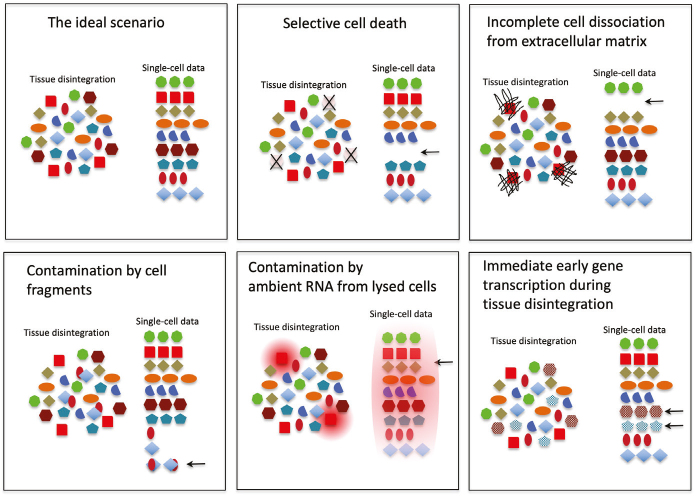
Confounders in single-cell RNA sequencing (scRNAseq) data. The cartoons illustrate the ideal scenario (top left), in which all cells of a tissue/organ become proportionally represented in a scRNAseq analysis with their *in vivo* transcriptome unperturbed. The subsequent panels illustrate various confounders that are all present to more or less extent in scRNAseq data. By recognizing the confounders, they may be handled to avoid data mis-interpretation.

It is well known that sudden changes to a cellular environment *in vitro* cause transcriptional activation of so-called immediate early genes (IEG), including the transcription factors Fos and Jun. ScRNAseq data show that this happens also during tissue dissociation into single cells (34), a process that requires enzymatic digestion for minutes-hours at physiological conditions, thereby allowing transcriptional changes to take place. Our tentative conclusion, based on available scRNAseq data, suggests that IEG activation occurs heterogeneously during tissue digestion. The proportion of IEG-activated cells seems to vary from experiment to experiment, and some cell types appear to be particularly prone to IEG responses, for example, arterial SMC and arterial endothelial cells. These cells are normally under physiological stress imposed by blood pressure and flow, and the sudden release of these physiological stresses in conjunction with animal euthanasia may be sufficient to induce an IEG response. Future analyses using conditions for rapid transcriptional silencing (freezing or placement in specific media) should further test these hypotheses. A summary of various confounders in scRNAseq data, including IEG gene activation and the contamination issues discussed above, is shown in Figure 9.

## Concluding remarks

With scRNAseq, vascular anatomy enters a new era – molecular anatomy – in which genome-wide quantitative transcriptional information may be retrieved for every vascular cell type, subtype, and state in the vasculature, at all stages of development and in all states of physiological and pathophysiological progression. The importance and usefulness of the type of encyclopedic molecular information that is obtained may not be fully appreciated until examples of direct utility have emerged, such as the identification of druggable vascular targets (we may recall analogous discussions at the start of the human genome project in 1990). A conservative guess is that the information will be of immense value. International consortium-led projects in the single-cell sphere (not limited to the vasculature) include the Human Cell Atlas (https://www.humancellatlas.org) and LifeTime (69).
